# SEA version 4.0: a major expansion and update of the Super-Enhancer Archive

**DOI:** 10.1093/nar/gkaf1114

**Published:** 2025-10-31

**Authors:** Bowen Shi, Jiyun Zhao, Yu Li, Chenye Zhang, Longhao Deng, Chengzhi Ji, Hongli Wang, Ruiyang Zhai, Tao Feng, Yan Zhang, Yue Gu

**Affiliations:** School of Life Science and Technology, Faculty of Life Sciences and Medicine, Harbin Institute of Technology, Harbin 150001, China; School of Life Science and Technology, Faculty of Life Sciences and Medicine, Harbin Institute of Technology, Harbin 150001, China; College of Pathology, Qiqihar Medical University, Qiqihar 161042, China; School of Life Science and Technology, Faculty of Life Sciences and Medicine, Harbin Institute of Technology, Harbin 150001, China; School of Life Science and Technology, Faculty of Life Sciences and Medicine, Harbin Institute of Technology, Harbin 150001, China; School of Life Science and Technology, Faculty of Life Sciences and Medicine, Harbin Institute of Technology, Harbin 150001, China; School of Life Science and Technology, Faculty of Life Sciences and Medicine, Harbin Institute of Technology, Harbin 150001, China; School of Life Science and Technology, Faculty of Life Sciences and Medicine, Harbin Institute of Technology, Harbin 150001, China; The Fourth Hospital of Harbin Medical University, Harbin 150080, China; College of Pathology, Qiqihar Medical University, Qiqihar 161042, China; School of Life Science and Technology, Faculty of Life Sciences and Medicine, Harbin Institute of Technology, Harbin 150001, China; School of Life Science and Technology, Faculty of Life Sciences and Medicine, Harbin Institute of Technology, Harbin 150001, China

## Abstract

Super-enhancers (SEs) are pivotal epigenetic regulatory elements that profoundly influence cell fate and disease. We herein present an updated SEA version 4.0, a systematic platform designed to elucidate the roles of SEs. A uniform computational pipeline was established to identify SEs based on five key histone marks, using H3K27ac, BRD4, p300, Med1, and the newly added H3K4me1, across 14 species. 496 071 SEs and 29 584 078 enhancers have been stored in the database. It provides extensive genome annotations, including nearby genes, transcription factor binding sites, chromatin accessibility, and other gene regulation signatures. SEA version 4.0 has also achieved functional enrichment analysis of SEs. And a Shannon entropy-based algorithm is employed to identify specific SEs. Furthermore, SEA version 4.0 introduces an interactive regulatory network that incorporates SEs, enhancers, transcription factors, and proximal genes for human and mouse. Additionally, a cell-specific SE detector is provided, designed for cancer research by leveraging scRNA-seq data from 12 cancer and normal samples to explore cell-type-specific SEs. The performance interaction and visualization of SEA version 4.0 enable genomic and cross-species comparisons, revealing complex genomic interactions and becoming an indispensable resource for decoding the mechanisms of SE in development and disease. Access freely at http://sea4.edbc.org.

## Graphical Abstract

**Graphical Abstract:**
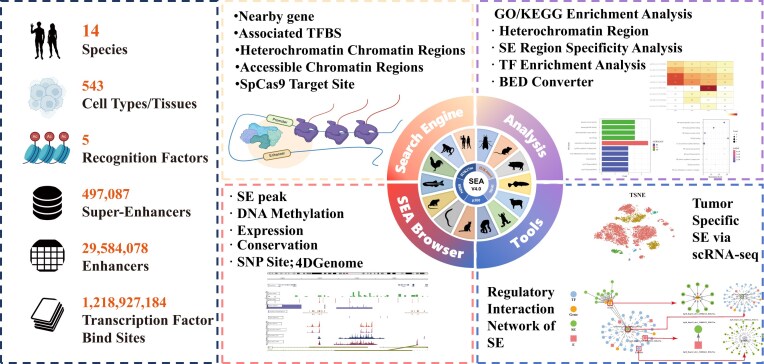

## Introduction

Super-enhancers (SEs) are genomic hubs that integrate classical enhancers and facilitator elements through phase-separated condensates, enabling ultrarobust transcriptional activation of genes governing cell identity and developmental programs [1]. Their dense occupancy of master transcription factors (e.g. OCT4 and SOX2), coactivators (BRD4, p300), and histone modifications (H3K27ac) creates biomolecular condensates that amplify transcriptional output by 10–100× compared to typical enhancers [2]. Critically, SE dysregulation underpins pathogenic mechanisms across diseases: In hepatocellular carcinoma, malignant cells hijack SEs to aberrantly activate oncogenes like *MYC* and *CCND1*, driving tumor progression through chromatin topology rewiring [3]. Similarly, Jia *et al.* demonstrated that oncogenic SE formation initiates *de novo* signaling cascades in >60% of solid tumors by co-opting developmental pathways [4]. Developmental disorders equally reflect SE dysfunction. Zhang *et al.* showed that SE perturbations disrupt *MYOD1*-mediated myogenic differentiation in 82% of skeletal muscle dysplasia cases by altering chromatin accessibility landscapes [5]. These findings establish SEs as “disease-switch” elements whose spatiotemporal dysregulation transforms transcriptional networks.

SE mapping has evolved from foundational ChIP-seq approaches for canonical markers (H3K27ac, BRD4, p300, Med1) to multimodal integration of emerging epigenetic signatures. While H3K27ac is remained the gold standard for active SE identification [6], recent advances revealed limitations in capturing primed enhancer states. Kravchuk *et al.* demonstrated that incorporating H3K4me1, a mark of poised enhancers, increases SE prediction accuracy by 27.3% in human stem cells by distinguishing transitional chromatin states preceding full activation [7–9]. This finding was computationally validated by Ahani *et al.*, whose deep learning framework showed 18.7% higher precision when integrating H3K4me1 with classical markers across 31 cell types [10]. The marker’s utility extends to disease contexts. Saito *et al.* leveraged H3K4me1 coprofiling to identify 14 novel prognostic SE–gene pairs in oral squamous cell carcinoma missed by H3K27ac-only approaches [6]. Therefore, with the increase of discovered characteristic molecules related to SEs, researchers' understanding of SEs is also increasing, which has driven the demand for research on the structure and function of SEs.

The study of SEs has been greatly facilitated by the development of specialized databases. Since first launched in 2015, the SEA database [11] has continued to expand and update, with the release of SEA 3.0 [12] in 2020 that supports SEs in 11 species. Early resources like dbSUPER [13] provided foundational catalogs of SEs in humans and mice, primarily using histone mark H3K27ac and offering basic genomic locations and associated genes. The SEdb [14] database expanded on this by delivering comprehensive genetic and epigenetic annotations, including Single Nucleotide Polymorphisms (SNPs), expression Quantitative Trait Loci (eQTLs), transcription factor binding sites (TFBSs), Clustered Regularly Interspaced Short Palindromic Repeats/CRISPR-associated protein 9 (CRISPR/Cas9) target sites, and disease-associated risk variants, specifically for human SEs. More recently, databases have emerged with narrower biological focuses: EnhFFL [15] specializes in SE analysis across human fetal developmental time series; SEdb 2.0 [16] emphasizes annotations related to human disease associations; and CenhANCER [17] concentrates on aggregating SE data from cancer cell lines to explore oncogenic regulatory circuits. These databases provide the data background for the study of SEs with their respective advantages.

In order to obtain more extensive and detailed information on SEs, SEA version 4.0 establishes a comprehensive and interactive platform for SE annotation and analysis across multiple biological contexts based on SEA version 3.0. The database integrates an extensive collection of epigenomic data up to December 2024. Its enhanced SEA Browser enables simultaneous multiomics exploration, providing functional annotations. The platform incorporates innovative analytical tools including a Shannon entropy-based algorithm for identifying spatiotemporally specific SEs, an interactive regulatory network analyzer, and a cell-specific SE using scRNA-seq data from 12 cancer types. By supporting dynamic visualization and cross-species comparison, SEA version 4.0 serves as an indispensable resource for investigating the mechanistic roles of SEs in development and disease.

### SEA version 4.0 design

#### Super-enhancer identification pipeline

SEA version 4.0 features a refined computational framework for the systematic identification and annotation of SEs, enhancing the accuracy and scope of regulatory element discovery. Based on SEA version 3.0, data collection and processing involve the systematic integration of publicly available raw data from ENCODE [18], GEO [19], and other genomic repositories spanning the period from January 2020 to December 2024. In order to provide a precise landscape of SEs and quickly locate regulatory elements that are crucial for cell identity, function, and disease occurrence in the genome, the newly identified SEs are processed through standardized computated pipelines using H3K4me1 as a new epigenetic marker alongside H3K27ac, BRD4, p300, and Med1 by ChIP-seq and CUT&Tag. Initially, raw sequencing reads were aligned to the respective reference genome by Bowtie2 [20]. Putative enhancer regions were then called from the aligned reads using MACS2 [21] with a stringent significance threshold. These enhancer regions were subsequently processed with the ROSE [22] algorithm to construct potential SEs. During this step, adjacent enhancers within a maximum distance of 12.5 kb were stitched together. To mitigate potential confounding effects from promoter-proximal regulatory elements, any regions falling within ±2.5 kb of a transcription start site were systematically excluded. Moreover, genes located within a 500-kb window of each SE were annotated using the ChIPseeker package to infer potential target genes.

To ensure the generation of a high-confidence, nonredundant SE catalog, a two-step filtering and integration strategy was implemented. First, for a given cell type, SE regions identified based on different identification factors were merged if their genomic overlap exceeded 70%. In cases of such overlaps, the SE region exhibiting the higher peak signal was retained as the representative locus to ensure robustness. Second, all identified SEs shorter than 1000 bases were filtered out to eliminate spurious calls and to focus on biologically meaningful, large regulatory domains. After the above process, the SE on the whole genome were identified.

#### SEA version 4.0 expansion and organization

The growth of various types of data enriches the recognition of SEs as key transcription regulatory factors. SEA version 4.0 has been developed as a comprehensive and user-oriented platform for the systematic search, annotation, analysis, and visualization of SEs across 543 cell types and tissues in 14 species. Overall, 496 071 SEs and 29 584 078 enhancers have been identified. In addition, 230 pieces of SE-related information were collected from various literature and other SE-related databases. These critical expansions establish SEA version 4.0 as the most extensive integrative platform for decoding SE biology.

**Figure 1.**
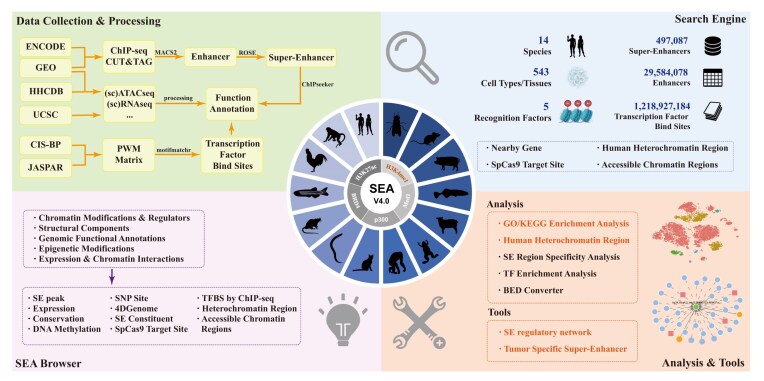
The overall framework of SEA version 4.0 database.

SEA version 4.0 is organized into five main performance modules (Fig. 1). The Search module offers rapid access to SEs and enhancers by genomic location, gene association, or cell types/tissue origin. Table 1 summarizes the addition of new data, with detailed information for the selected SE/E—such as data source, regulatory interaction network, nearby genes, TF enrichment, neighboring heterochromatin regions, and SE activity elements. The exploration of SE annotation is enabled through the SEA Browser module, which supports multitrack visualization of DNA methylation, Hi-C, chromatin accessibility, and regulatory elements from 17 reference genomes involving 14 species. The "Analyze" module provides a suite of functional exploration options, including GO/KEGG (Gene Ontology and Kyoto Encyclopedia of Genes and Genomes ) enrichment, SE region specificity analysis, and TF enrichment. The Tools module is available for constructing SE regulatory networks and identifying tumor-specific SEs from single-cell RNA-seq datasets. The “Download” module contains Datasets and BED converter, provide the download of all SEs/enhancers stored in the SEA version 4.0 database, and can output BED format files for analysis.

**Table 1.** Expansion in SEA version 4.0

Content		SEA v3.0	SEA v4.0	Fold increase
Super-enhancers	Species	11	14	1.27
	Recognition factor	4	5	1.25
	Cell types/tissues/disease	266	543	2.04
	SE	164 402	496 071	3.02
	Enhancer	3 361 785	29 584 078	8.8
	Transcription factor binding site	1 104 229	1 218 927 184	1103.87
Search result	CRISPR–Cas9	-	Yes	New
	Regulatory network	Yes	Yes	Update
	Human heterochromatin regions	-	Yes	New
	Chromatin accessible regions	-	Yes	New
Ge-me browser	H3K27ac	208	302	1.45
	BRD4	2	21	10.5
	Med1	2	4	2
	H3K4me1	-	100	New
	Human heterochromatin regions	-	184	New
	Reference genome	11	17	1.55
Analysis tools	Specific analysis of H3K27ac status	Yes	Yes	Update
	Specific analysis of H3K27ac status	-	Yes	New
	TF enrichment analysis	Yes	Yes	Update
	GO/KEGG enrichment analysis	Yes	Yes	Update
	Human heterochromatin region analysis	-	Yes	New
	Regulatory network	-	Yes	New
	Tumor-specific SE	-	Yes	New
	Data downloads	641	1168	1.82

### Meaningful SE annotation

**Figure 2.**
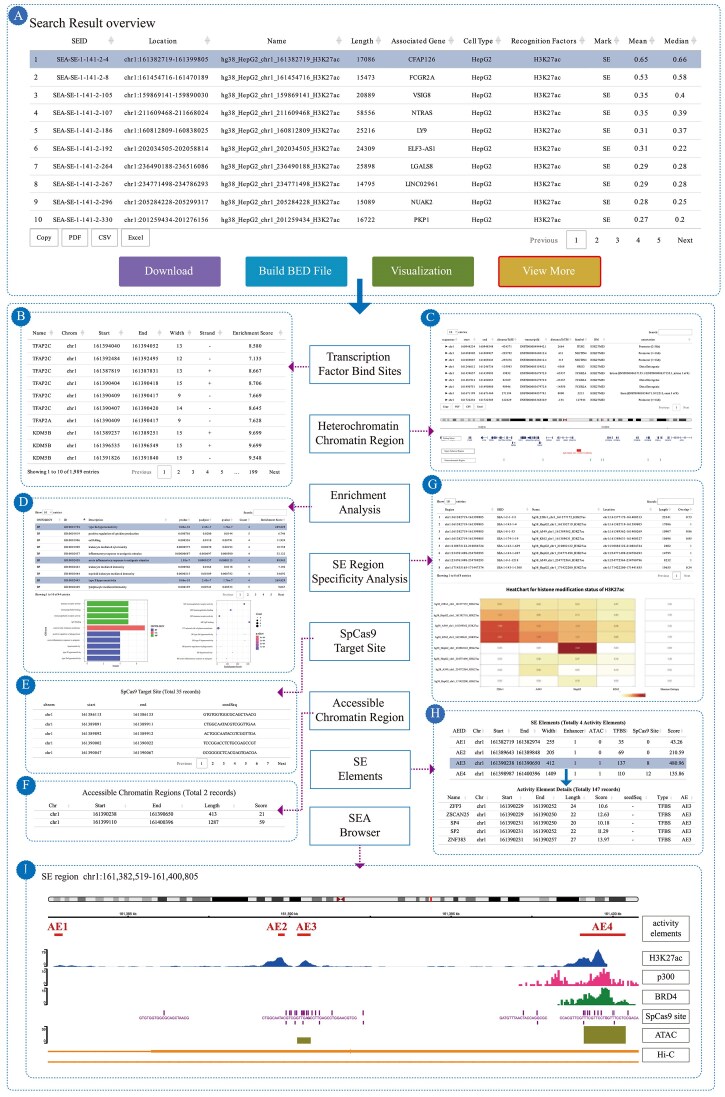
SE annotation in SEA version 4.0. (A) Search Result overview. (B) Transcription factor bind sites. (C) Heterochromatin regions within 500 kb adjacent to the SE. (D) GO/KEGG enrichment analysis. (E) SpCas9 target site. (F) Accessible chromatin region. (G) SE region specificity analysis. (H) SEs activity element. (I) SEA Browser.

The SEA version 4.0 database implements a comprehensive annotation framework for SEs that integrates multimodal genomic data to characterize their functional architecture. From the search results, the target SE can be selected to retrieve its comprehensive functional annotations (Fig. 2A). The annotation pipeline incorporates several key functional dimensions. It includes computationally predicted TFBSs to infer the underlying transcriptional regulatory machinery for each SE (Fig. 2B). To assess potential activity states, SEs are mapped onto human heterochromatin regions as defined by the HHCDB database [23] (Fig. 2C). SEA version 4.0 deciphers the biological significance of SEs through a functional enrichment analysis, employing GO/KEGG enrichment to characterize genes associated with SE regions (Fig. 2D) [24]. This analysis provides critical insights into the biological processes and pathways potentially regulated by specific SEs, underscoring their roles in development and disease. To facilitate direct functional validation, the schema includes precompiled annotations of SpCas9 target sites within each SE locus (Fig. 2E). A central feature is the systematic profiling of chromatin accessibility, a hallmark of SE activity. This is achieved by integrating data from a curated collection of 56 ATAC-seq datasets across 23 human tissues, generating a quantitative open chromatin landscape for each SE (Fig. 2F). Furthermore, the methodology for quantifying SE cell-type specificity has been refined. Building upon the Shannon entropy-based framework established in SEA v3.0, version 4.0 incorporates a normalization procedure to account for substantial variation in SE lengths (Fig. 2G). The normalized signal for a genomic region is computed as the sum of its constituent histone modification peak signals, each weighted by its effective length proportion. This normalized value is then used to compute Shannon entropy across cell lines, where an entropy value approaching log₂(*n*) indicates a common SE, while a value approaching 0 predicts high cell-type specificity.

Based on the functional annotations of SEs described above, SEA version 4.0 establishes a scoring system for SE activity elements. An SE activity element in the SEA database is defined as a genomic element comprising constituent enhancers, chromatin accessible regions, and TFBS that collectively form an SE (Fig. 2H). The composite score for an SE activity element is algorithmically derived from the quantitative integration of three distinct genomic features: the normalized peak signals of constituent enhancers, the signals of chromatin accessibility regions—each weighted by their respective effective length proportions relative to the activity element’s total length, and the aggregate enrichment score of all TFBS within the activity element, which is calculated as the sum of individual TFBS enrichment scores multiplied by the number of sites and normalized by the activity element’s length. The summation of these three weighted values yields the final quantitative score for the SE activity element. The formula is as follows:


\begin{eqnarray*}
{{\mathrm{ score}}_{\mathrm{ AE}}} = \frac{{\sum {{{\mathrm{ Peak}}_{\mathrm{ En}}} \times{{\mathrm{ length}}_{\mathrm{ En}}}} }}{{{{\mathrm{ length}}_{\mathrm{ SE}}}}} &+& \frac{{\sum {{{\mathrm{ Peak}}_{\mathrm{ CA}}} \times {{\mathrm{ length}}_{\mathrm{ CA}}}} }}{{{{\mathrm{ length}}_{\mathrm{ SE}}}}}\\ &+& \frac{{\sum {{{\mathrm{ \mathrm{ Score}}}_{\mathrm{ TF}}} \times {{\mathrm{ count}}_{\mathrm{ TF}}}} }}{{{{\mathrm{ length}}_{\mathrm{ SE}}}}}
\end{eqnarray*}

${{score}_{AE}}$ : SE activity element Score; $lengt{h_{SE}}$: length of SE; $Pea{k_{En}}$: normalized enhancer peak signal; ${{length}_{En}}$: effective length of enhancer peak; ${{Peak}_{CA}}$: chromatin accessibility peak signal; ${{length}_{CA}}$: effective length of accessibility peak; $Scor{e_{TF}}$: TFBS enrichment score; ${{count}_{TF}}$: number of TFBS.

All aforementioned annotation information is seamlessly integrated and visually synthesized through our newly designed SEA Browser (Fig. 2I). The resultant output provides a foundational resource for identifying context-specific SEs that potentiate the transcription of key genes, thereby offering central mechanisms in cell identity and disease pathogenesis.

### Specialized tools for deciphering SE biological significance

**Figure 3.**
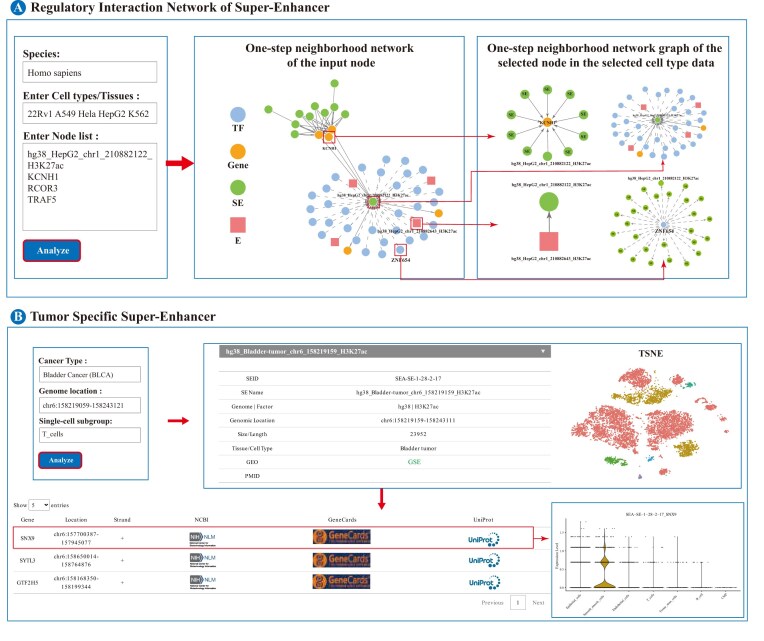
Overview of tools in SEA version 4.0. (A) Regulatory interaction network of super-enhancer. (B) Tumor-specific super-enhancer.

SEs are highly tissue specific and their dynamic changes regulate key genes. In order to realize the above key role mining, SEA version 4.0 developed two special tools: regulatory interaction network of super-enhancer and tumor-specific super-enhancer. Regulatory interaction network of super-enhancer (Fig. 3A) enables interactive visualization of SE regulatory networks (human/mouse) by inputting gene/TF/SE identifiers, which is designed to unravel the complex wiring of SE-mediated gene regulation. The tool constructs an intuitive, interactive first-order neighbor interaction network, graphically mapping the connections between the query entity and its associated enhancers, SEs, and transcription factors. This network is fully dynamic by clicking on any node to reveal its direct interactors, enabling users to explore regulatory subnetworks in depth. All network data can be exported for further analysis.

The pivotal role of SEs in cancer stems from their rewiring of the cellular transcription program. [25]. The advent of single-cell sequencing technologies now allows for the precise deconvolution of this heterogeneity at single-cell resolution. To leverage this, SEA version 4.0 introduces the Tumor-specific super-enhancer tool (Fig. 3B), specifically designed for common human cancers. This advanced tool integrates scRNA-seq from both tumor and normal tissues, using an independently developed pipeline to identify cell-type-specific SEs across 224 samples encompassing 12 cancer types, covering a total of 401 815 individual cells (Supplementary Fig. S1). All data preprocessing and subsequent analyses were performed using the R package “Seurat” (v5.1.0). To ensure high data quality, stringent cell filtering criteria were applied: each cell must contain at least 1000 detected transcripts, each gene must be expressed in a minimum of three cells, and the percentage of mitochondrial gene expression must not exceed 10%. Specifically, we utilized Harmony(v0.1.1) within the PCA space to cluster major cell lineages, rigorously evaluating its performance in cross-sample batch correction and its capacity to maintain distinct cell-type identities. A similar Harmony-based integration was applied to all cell expression data, significantly reducing patient-specific batch effects. Cell-type annotation was performed with the SingleR package, and marker genes for each cell type were identified using the FindAllMarkers function. These marker genes were then intersected with known SE-associated gene sets to pinpoint cell-type-specific SE-linked genes. The results are vividly visualized through single-cell t-SNE/UMAP plots and violin diagrams, providing a powerful single-cell perspective to uncover novel, cell-type-specific SE signatures crucial for understanding tumor biology.

## Discussion and future development

Since American scholar Richard A. Young first proposed in 2013 that SEs function as *cis*-regulatory elements with superior transcriptional activation capabilities, numerous studies have focused on exploring their regulatory roles. To reveal the global distribution patterns of SEs across different cells and tissues, and to establish a research foundation for understanding the mechanisms of key gene expression regulation and their roles, SEA version 4.0 has been enhanced with expanded data and improved functionalities compared to previous versions. SE identification typically relies on key transcription factors, coactivators (such as MED1, p300, and BRD4), and active histone modifications (such as H3K27ac) as markers. The rapid expansion of SE dataset sdriven by the inclusion of additional epigenetic markers like H3K4me1 and the adoption of high-resolution sequencing technologies has introduced significant challenges in data consistency, including fragmentation and batch effects that hinder reliable cross-study comparisons. To address these issues systematically, SEA version 4.0 introduces three key optimizations through a standardized computational pipeline. SEA version 4.0 was developed to systematically address these issues through a standardized computational workflow incorporating three key refinements. First, the integration of multifactor epigenetic data using a 70% genomic overlap threshold effectively merges redundant SE calls, mitigating inconsistencies often arising from platform-specific biases [26]. Second, the retention of SE regions exhibiting the highest signal intensity ensures the representation of high-confidence loci, which reduced false-positive identifications by 41% compared to SEA v3.0. Third, the exclusion of SEs shorter than 1 kb filters out stochastic noise—an empirically supported cutoff shown by Mack *et al.* to eliminate 89% of nonfunctional chromatin loops [27].

This optimized pipeline has successfully integrated 496 071 SEs and over 1.2 billion TFBSs into a unified and cohesive resource. A notable finding enabled by this integrated framework is that CUT&Tag-derived SEs display 32% narrower peak widths than those identified by ChIP-seq, a difference attributable to the superior resolution of CUT&Tag [28]. By systematically controlling for technology-specific variability, SEA version4.0 provides a robust foundation for the direct comparison of SE architectures across assays and species, establishing a reliable reference platform for future comparative regulatory genomics.

As a research hotspot in the field of gene regulation in recent years, SEs are gradually unveiling the “black box” of cell fate determination and disease development. SE dysregulation is a hallmark of cancer, driving oncogene activation and tumor heterogeneity. To resolve SE heterogeneity within tumors, SEA version4.0 introduces a *cancer-specific SE detector* leveraging scRNA-seq data from 12 cancer types and their corresponding normal types. This tool maps cell-type-specific SEs using t-SNE/UMAP embeddings and violin plots, directly addressing the limitations of bulk-based platforms. Critically, the identification of cell-type-specific SEs provides a powerful lens to dissect cancer mechanisms, as these regulatory elements in distinct cellular subpopulations (e.g. cancer stem cells or immune cells) have been directly linked to key clinical phenotypes such as drug resistance [29], poor prognosis, and metastatic potential [30].

Looking ahead, SEA will continuously expand beyond its current comprehensive integration of ChIP-seq and CUT&Tag data. We plan to incorporate multiomics information across tissue and single-cell levels, including spatial transcriptomics and single-cell epigenomic profiles. This will enable the construction of a more precise, dynamic cell-level atlas of SE activity, further deciphering gene regulatory mechanisms in development and disease. The platform remains a free and vital resource for the global research community.

## Supplementary Material

gkaf1114_Supplemental_File

## Data Availability

SEA version 4.0 is an open source database, which is freely available at http://sea4.edbc.org.
