# Supplementary material for: SEA version 4.0: a major expansion and update of the Super-Enhancer Archive
Source: Nucleic Acids Res. 2025 Oct 31;54(D1):D314–21. doi: 10.1093/nar/gkaf1114 (PMC12807652; doi:10.1093/nar/gkaf1114)
Supplement: gkaf1114_Supplemental_File [file gkaf1114_supplemental_file.pdf]

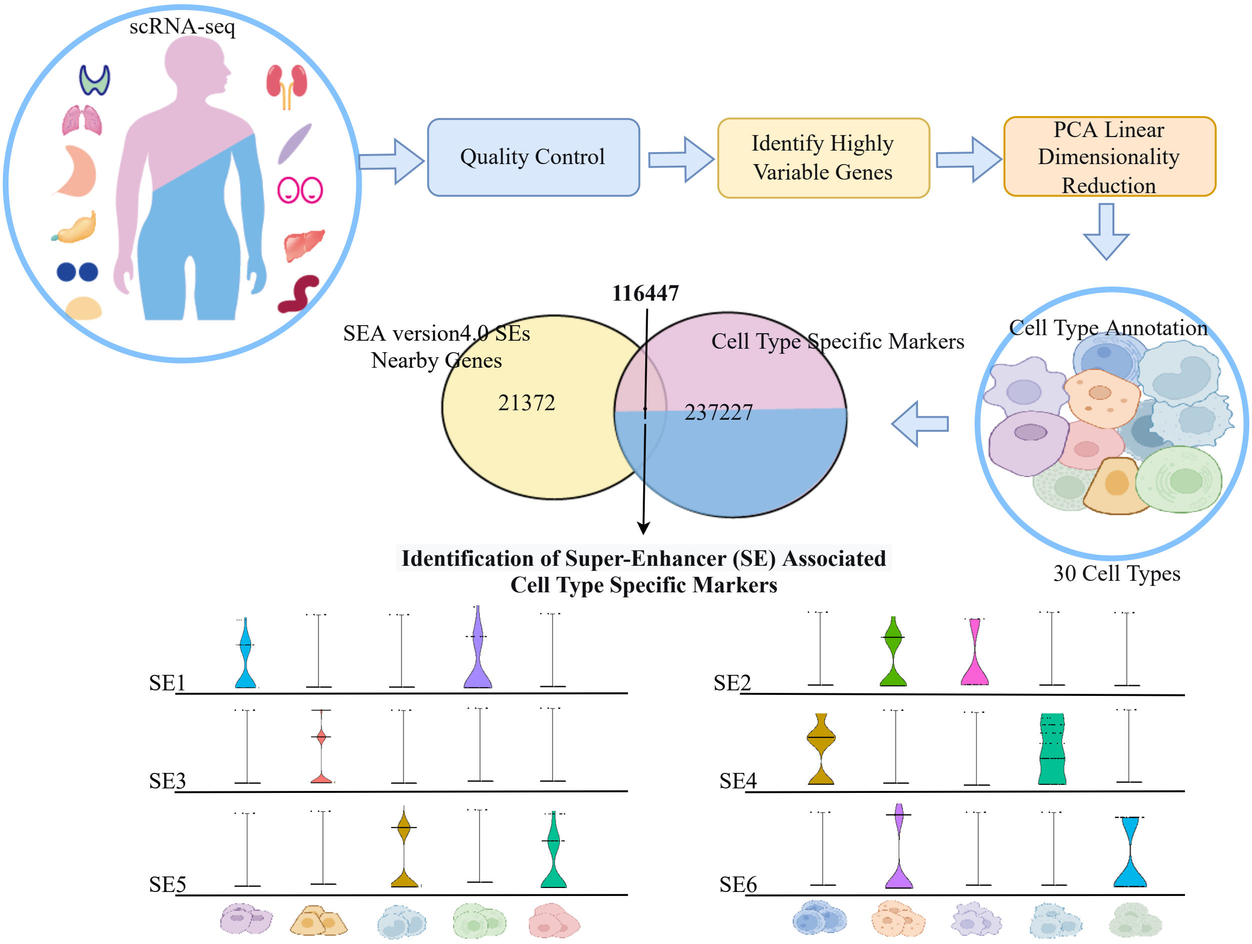

Supplementary Figure 1, Workflow for identifying cell-type-specific super-enhancers from scRNA-seq data.
